# Iodized household salt utilization and associated factors among households in East Africa: a multilevel modelling analysis using recent national health surveys

**DOI:** 10.1186/s12889-023-17296-x

**Published:** 2023-12-01

**Authors:** Bewuketu Terefe, Mahlet Moges Jembere, Nega Tezera Assimamaw

**Affiliations:** 1 Department of Community Health Nursing, School of Nursing, College of Medicine and Health Sciences, University of Gondar, Gondar, Ethiopia; 2 Department of Emergency and Critical Care Nursing, School of Nursing, College of Medicine and Health Sciences, University of Gondar, Gondar, Ethiopia; 3 Department of Pediatric and Child Health, School of Nursing, College of Medicine and Health Sciences, University of Gondar, Gondar, Ethiopia

**Keywords:** Iodized household salt utilization, Factors, Households, East Africa, Multilevel model

## Abstract

**Introduction:**

Iodine deficiency disorders (IDDs) are a significant global public health issue that affects the physical and mental development of every age group, with children and nursing mothers being the most vulnerable. Approximately 50 million of approximately 2 billion people with iodine deficiency (ID) globally exhibit clinical symptoms. Identifying iodine levels using various techniques is important when considering treatment choices. Screening programs and early ID diagnostics are crucial for the follow-up of pregnant women, especially in iodine-deficient nations. There have been calls for universal salt iodization programs, but only approximately 71% of people have access to them. The problem is more common in developing nations; however, there is a shortage of literature on the individual-, family-, and community-level factors influencing iodized salt use in East Africa. This study aimed to investigate individual- and community-level factors of household iodized salt usage in East Africa.

**Methods:**

Using Stata 17, this study used the most recent demographic and health survey datasets from twelve East African countries. The survey included a weighted sample of 154,980 households. To assess factors related to iodized salt use in the region, bivariable and multivariable multilevel logistic regressions were used. P values less than or equal to 0.2, and < 0.05 were used in the binary regression, and to deem variables statistically significant in the final model respectively.

**Results:**

About 87.73% (95% CI = 87.56,87.89) households have utilized iodized household salt. Secondary and above education (AOR = 1.23, 95% CI = 1.17–1.30), household heads with ages of 25–35 years, 36–45 years (AOR = 1.20, 95% CI = 1.12,1.28), 36–45 years (AOR = 1.16, 95% CI = 1.09,1.24), and more than 45 years (AOR = 1.18, 95% CI = 1.11,1.25), lower and middle wealth (AOR = 0.89, 95% CI = 0.76,0.89) and (AOR = 0.97, 95% CI = 0.81,0.93), media exposure (AOR = 1.10, 95% CI = 1.07–1.14), female household leaders (AOR = 1.08, 95% CI = 1.04–1.12), access to improved drinking water and better toilet facility (AOR = 2.26, 95% CI = 2.18–2.35) and (AOR = 1.50, 95% CI = 1.44,1.56), larger than five family members (AOR = 0.96, 95% CI = 0.93–0.99), high community level wealth (AOR = 1.54, 95% CI = 1.27–1.87), and low community education(AOR = 0.40, 95% CI = 0.33,0.49) were statistically associated with utilization of iodized household salt in East Africa respectively.

**Conclusion:**

In East Africa, household salt iodization is moderately good. To expand the use of iodized salt in the region, access to improved drinking water and toilet facilities, participating family leaders, using the opportunity of family planning services, media sources, and the improvement of the community’s socioeconomic level are all needed.

## Introduction

The iodine element is essential for the synthesis of thyroid hormone (TH). This hormone influences the body’s ability to grow, reproduce, and control its metabolic processes [1, 2]. Iodine deficiency diseases (IDDs) arise when the body does not receive the required amount of iodine daily. IDDs may also result in mental disability, endemic goiter, hypothyroidism, infertility, and dwarfism [2, 3]. The most affordable, frequently utilized, and low-cost public health strategy for providing the necessary amount of iodine to the general population is salt iodization [2, 4]. The most significant factor contributing to mental impairment that can be avoided through routine iodization and other public health measures is iodine deficiency [5]. Sub Saharan Africa, including East African countries are among the top sub-Saharan countries in terms of the availability of non-iodized salt in households, and many African nations do not achieve at least 90% universal salt iodization [6, 7].

IDDs are major public health problems that impact more than 50 different countries. According to World Health Organization (WHO) estimates, there are roughly 2 billion people globally [8]. Iodine deficiency affects 30% [9] of the global population, or those who have blood iodine levels below 100 g/L [10]. Globally, iodine deficiency is a hazard for public health [11, 12]. The countries most impacted are those in Europe (57%) as well as the Eastern Mediterranean (54%), Africa (43%), Southeast Asia (40%), the Western Pacific (24%), and the Americas (10%) [13, 14]. Iodine deficiency states, which may be linked to a 10–15% decline in average intellectual capacity, are present in roughly 260 million people in Africa [15, 16]. This lack of iodine in food crops causes an iodine deficiency in the diet. People therefore require additional sources in order to ingest the necessary levels [17]. Nevertheless, the Universal Salt Iodization (USI) project, a highly cost-effective public health policy, was backed by the WHO [18], and salt iodization campaigns were launched in about 120 different countries globally. Iodine deficiency disorders have been eliminated in 34 of these nations as a result of USI [3].

Less than 10% of the world’s populace in 2017 resided in nations that were classified as having an iodine deficiency [19]. IDDs affect people of all ages, but young children, women who are pregnant, nursing, or of reproductive age are especially at risk [3]. IDDs are still a problem for public health [20]. Iodine intake recommendations from the World Health Organization (WHO) are 150 g/day for adults and adolescents 13 years of age and older, 200 g/day for women who are pregnant or nursing, 120 g/day for children 6–12 years of age, and 90 g/day for infants up to 59 months of age [21].

Evidences suggest that the availability of iodized salt varies by country, with Bangladesh having a rate of 73.15% [22], and Saudi Arabia having a rate of 69.8% [23]. The percentage of families using iodized salt in African countries ranges from 6.2% in Niger to 97% in Uganda [24], which is lower than the global recommendation of > 90% of households using iodized salt [2]. More than 10% of families in Ethiopia don’t use iodized salt [7]. In Bangladesh, the availability of iodized salt in households is dramatically increased and decreased by young, educated household heads, impoverished, and rural families [22]. In different nations, the lack of iodized salt is correlated with various socioeconomic and household-level characteristics. From past studies, variables such the household head’s age and education level, wealth index, place of residence, awareness of iodized salt, marital status, family size, and use of packed salt were considered [7, 25–27]. Iodized salt availability in East Africa varied significantly by region or countries ranging from 41.8 to 95%, as shown by systematic review, and national health survey reports [25, 28, 29]. However, the pooled prevalence, and its associated factors in East Africa was not addressed by these investigations. Thus, the current study will provide an answer to this question by considering these and other potential variables. The study will also highlight the region’s present use of iodized salt so that policymakers may base their decisions on it. Therefore, this study was designed to assess the pooled prevalence of household iodized salt, and its associated factors among East African household members using recent national health survey data. to achieve the universal salt iodization.

## Methods

### Study setting, and period

The most recent standard Demographic and Health Survey (DHS) dataset for East African nations during a ten-year period (2012–2022) provided the information for this study. A standardized data set was employed [30] To collect a sizable sample that is representative of the population source and all factors. DHS gathers comparable data on a global scale using a nationwide cross-sectional study design every five years. The surveys have huge sample sizes, are population-based, and nationally representative of each nation [30]. The 14 nations that make up Eastern Africa are spread throughout the Horn of Africa, the Indian Ocean islands, and the Great Lakes region. These nations struggle with low economic, social, and environmental problems, and they worry that they won’t achieve all of the Millennium Development Goals’ objectives [31]. East Africa is the portion of the African continent that lies in the horn and eastern parts of the Sahara Desert. They are estimated to be home to 486,766,759 people and cover an area of 6,667,493 Km2 (2,574,332 square miles), making up 6.03% of the world’s population [31].

### Data source and study population

More than 90 low-income and middle-income nations were included in the data collection of the DHS program. The data collection for each nation was comparable. The program used the same manual, data collection instrument, variable name, variable code, and sampling techniques. We used the DHS completed over the last ten years, from 2012 to 2022. Although around 14 East African nations conducted DHS between 2012 and 2022, only about 12 countries’ DHS were used for this analysis because the remaining surveys lacked information on the outcome variable. After each country’s data were incorporated, a total weighted sample of 154,980 households and an unweighted sample of 154,732 households who had been interviewed for iodination of salt were used for the final analysis (Table 1).

**Table 1:** Countries, sample size, and survey year of Demographic and Health Surveys included in the analysis for 12 East African countries

Country	Survey year	Sample size(weighted)	Frequency(weighted)
Burundi	2016/17	14,389	9.28
Ethiopia	2016	15,939	10.28
Kenya	2022	36,002	23.23
Comoros	2012	3,892	2.51
Malawi	2016/17	22,489	14.51
Mozambique	2011	13,140	8.48
Tanzania	2015	11,737	7.57
Uganda	2016	17,851	11.52
Zambia	2018	11,485	7.41
Zimbabwe	2015	8,057	5.20

### Sample size determination and sampling method

Demographic and Health Survey Reports were available for about 12 of East Africa’s 13 nations. Each of the surveys conducted in the countries below (Table 1) used the most recent conventional census frame. Within each administrative geographic region, the DHS samples are usually separated into urban and rural areas. A stratified, multistage, random sample design was used for the survey. Enumeration Areas (EA) were initially selected by each nation’s census files. Second stage: A sample of households is chosen from an updated list of households in each EA that has been chosen. Data were gathered from qualified individuals in each nation. Other sources have described the survey methodology and sample techniques used to collect the data in detail. Enumeration areas (EAs) were chosen with a probability proportional to their size within each stratum during the first round of sampling. In the second sampling step, the systematic sampling approach selects a specified number of households in the designated EAs. Following the listing of households, equal probability systematic sampling is used to select a specific number of households from inside the defined cluster [30].

### Data collection tools and procedure

Every five years, the DHS collects nationally representative data and reflects the demographic and health challenges unique to each nation using five surveys. These included questionnaires for households, women, and men, biomarkers, and health institutions. The household questionnaire used in this study ascertained the regional use of iodized salt and its contributing factors. As a result, this investigation was conducted in accordance with the pertinent DHS statistical rules. To acquire data that are comparable across nations, the DHS program uses standardized methods that involve uniform questionnaires, manuals, and field procedures.

### Data quality control

In the DHS, a pre-test was conducted before data collection, a debriefing session with pre-test field workers was held, and changes to the questionnaires were made as necessary. The DHS guidance provides additional details regarding the data-collection process. Details can be accessed from the Guide to DHS statistics.

### Variables of the study

#### The outcome variable

The outcome variable was percentage of households with salt tested for iodine content, with salt tested among households tested percentage with iodized salt. Then the outcome variable was recategorized as Yes = “1” if the household received iodized household salt, and No= “0”, if the household did not receive it. This classification, and the analysis has been made according to the guide to DHS statistics book [32].

#### The independent variables

Independent variables: household level and sociodemographic related factors were included. This included household age (< 25, 25–35, 36–45, and > 45 years old), sex (male/ female), educational status (not formally educated, primary, secondary/higher), types of places of residence(urban/rural), marital status (not married/married), household wealth index (poorest, poorer, middle, richer and richest), current employment status (employed/not employed), mass media exposure (yes/no), household member (up to five, and greater than five), source of drinking water (improved/not improved), toilet facility types (improved/not improved), community level literacy (low/high), country, and community level wealth (low/high) were included.

### Data management and statistical analysis

STATA version 17 was used to extract, clean, and recode the variables in the study. The data were weighted using sample weight during any statistical analysis to account for the differential probability of selection due to the sampling procedure used in DHS data. As a result, the survey results were ensured to be representative. To account for the hierarchical nature of the data, a two-level multilevel fixed effect binary and multiple logistic regression analysis were employed to evaluate the effect of explanatory variables on the salt iodization among households in east Africa. The data is divided into two levels, with a group of J EAs and within-group j (j = 1, 2…J), and a random sample nj of level-one units (households). The response variable is represented as; Y_ij_ = 0 if the i^th^ households was in the j^th^ EAs had an exposure of salt iodization 1 if i^th^ households was in the j^th^ EAs had no exposure of salt iodization consumption.

To account for the nested effect, acceptable deductions and conclusions from this data require adequate modeling techniques such as multilevel modeling, which includes variables assessed at multiple levels of the hierarchy [33]. Four models were fitted to the data. The initial model used to calculate the extent of cluster variation in salt iodization was an empty model with no explanatory parameters. To compute differences between clusters (EAs), the intra-class correlation coefficient (ICC), proportional change in variance (PCV), and median odds ratio (MOR) were employed. The fraction of variance explained by the population grouping structure is represented by the ICC. Unlike the null model, PCV assesses the total variation attributable to individual and community level components in the multilevel model [34].

When two clusters (EAs) are picked at random, the MOR is defined as the median value of the odds ratio between the clusters at high and low risks of salt iodization. The second model included only community-level variables, the third model included only individual-level variables, and the fourth model included both individual- and community-level variables. The model with the lowest deviance (-2LLR) was chosen as the best-fit model for the data. Regarding the model parameter estimation for this investigation, a generalized linear mixed model (GLMM) was utilized, in which both random and fixed effect analyses were included in the linear predictor. Variables with a p-value of less than or equal to 0.2 in the bivariate analysis were considered for the multivariable analysis. In the multivariable multilevel binary logistic model, the best-fit model’s Adjusted Odds Ratio (AOR) with 95% CI was reported to determine the factors associated with salt iodization among households. The statistical significance of the final model was set at a p-value < 0.05. Before proceeding to the analysis, each dependent variable was assessed for its variance, inflation factors, and tolerances. The mean VIF in this study was 1.22.

## Results

### Sociodemographic characteristics of study participants on individual and community

This survey comprised weighted samples from 154,980 households. In terms of age, approximately 61,581 (39.73%) household heads were over 45 years. The majority, 111,953 (72.40%), and 110,471 (71.28%), were married and lived in rural areas, respectively. In terms of educational level, 69,458 (44.86%) participants had completed primary school. Media exposure was reported by 87,902 (56.72%) participants. Approximately 106,921 (68.99%) households were headed by males. In terms of drinking water source and toilet facility types, the majority 116,039 (74.87%) and almost half 81,854 (52.82%) of them had upgraded drinking water sources and toilet facilities, respectively. The majority of households, 105,373 (67.99%), had fewer than six family members. About 33,858(21.85%) households revealed the richest wealth quantile. (Table 2).

**Table 2:** Sociodemographic, and household related characteristics of iodization of household salt among households in east Africa (unweighted n = 154,589, and weighted = 154,980)

Variables on iodization of household salts	Frequency	Percentage
**Household head age**		
> 25	11,721	7.56
25–35	45,425	29.31
36–45	36,254	23.39
> 45	61,581	39.73
**Marital status**		
Not married	42,676	27.60
Married	111,953	72.40
**Educational status**		
No formal education	36,292	23.44
Primary	69,458	44.86
Secondary and higher	49,089	31.70
**Household wealth index**		
Poorest	28,738	18.54
Poorer	29,625	19.12
Middle	30,111	19.43
Richer	32,649	21.07
Richest	33,858	21.85
**Mass media exposure**		
No	67,079	43.28
Yes	87,902	56.72
**Sex of the household head**		
Male	106,921	68.99
Female	48,059	31.01
**Source of drinking water**		
Unimproved	38,941	25.13
Improved	116,039	74.87
**Toilet facility type**		
Unimproved	73,126	47.18
Improved	81,854	52.82
**Household member**		
1–5	105,373	67.99
> 5	49,607	32.01
**Types of residence**		
Urban	44,510	28.72
Rural	110,471	71.28
**Community-level wealth**		
Low	79,082	51.03
High	75,898	48.97
**Community illiteracy**		
Low	80,187	51.74
High	74,793	48.26

### Random effect analysis

The random-effects model revealed considerable clustering of iodized salt usage across communities (OR of community-level variance = 2.19). The ICC value in the null model revealed that cluster variability accounted for 40.00% of the overall variation in the iodized salt use. The null model’s 4.08 MOR value also implies that there is variance in iodized salt use among the clusters. This means that if we randomly selected houses from different clusters, those in the cluster with the highest household iodized salt utilization had a 4.08 times greater likelihood of utilizing iodized salt than their counterparts did. The PCV increased from 9.59% (Model I) to 11.42% (Model III), as shown in Table 3, demonstrating that Model III best describes the heterogeneity of iodized salt usage. Furthermore, Model III had the lowest Deviance and AIC. Therefore, it was chosen as the best-fitting model (Table 3).

**Table 3:** Individual and community-level factors associated with iodization of household salt among households in east Africa (unweighted n = 154,589, and weighted = 154,980)

Variables on iodization of household salts	Null model	Model I	Model II	Model III
		AOR (95% CI)	AOR (% CI)	AOR (95% CI)
**Household head age**				
> 20		1		1
20–35		1.20(1.33,1.75)		1.20(1.12,1.28) *
36–45		1.16(1.31,1.73)		1.16(1.09,1.24) *
> 45		1.19(1.34,1.77)		1.18(1.11,1.25) *
**Educational status**				
No formal education		1		1
Primary		1.01(0.96,1.04)		1.01(0.96,1.04)
Secondary and higher		1.19(1.13,1.26)		1.23(1.17,1.30) *
**Household wealth index**				
Poorest		1.26(1.18,1.35)		0.85(0.89,1.04)
Poorer		1.07(1.01,1.14)		0.89(0.76,0.89) *
Middle		1.08(1.03,1.16)		0.97(0.81,0.93) *
Richer		1.11(1.04,1.17)		1.01(0.94,1.09)
Richest		1		1
**Mass media exposure**				
No		1		1
Yes		1.10(1.06,1.15)		1.10(1.07,1.14) *
**Sex of the household head**				
Male		1		1
Female		1.06(1.02,1.10)		1.08(1.04,1.12) *
**Source of drinking water**				
Unimproved		1		1
Improved		2.19(2.11,2.26)		2.26(2.18,2.35) *
**Toilet facility type**				
Unimproved		1		1
Improved		1.49(1.43,1.54)		1.50(1.44,1.56) *
**Household member**				
1–5		1		1
> 5		0.97(0.94,1.01)		0.96(0.93,0.99) *
**Types of residence**				
Urban			1	1
Rural			0.88(0.85,0.92)	0.92(0.90,1.01)
**Community-level wealth**				
Low			1	1
High			1.44(1.18,1.76)	1.54(1.27,1.87) *
**Community illiteracy**				
Low			1	1
High			0.36(0.29,0.44)	0.40(0.33,0.49) *
**Random parameters and model comparison**				
Community-level variance	2.19	1.98	2.03	1.94
ICC (%)	40.00	37.59	38.17	37.30
MOR (%)	4.08	3.81	3.87	3.76
PCV	Reference	9.59	7.31	11.42
Log-likelihood (LLR)	-53603.4	-51985.8	-53524.5	-51797.4
DIC (-2LLR)	107,206.8	103,971.6	107,049.0	103,594.8
AIC	107210.7	104003.7	107059.1	103638.8

*Indicates significance at p-value < 0.05 variables in the regression

### Associated factors of iodized salt utilization among households in East Africa

Bivariable and multivariable multilevel logistic regression analyses were used to identify factors associated with iodized salt use. Accordingly, in the binary model analysis, the age and gender of the household head, educational level, marital status, wealth index, media exposure, source of drinking water, toilet facility type, family size, type of residence, community wealth, and community level were all associated or selected as candidate variables for the final model with iodized salt use in East Africa (P < 0.2). The final model indicated that household head’s age and gender, media exposure, wealth index, community education, education level, source of drinking water, types of toilets, number of family members, and community wealth were significantly linked with iodized salt use (p < 0.05).

Households with secondary/higher education had 1.23 times the chances of using iodized salt compared to those with no formal education (AOR = 1.23, 95% CI = 1.17–1.30). Households with ages of 25–35 years, 36–45 years, and more than 45 years had 1.20, 1.16, and 1.18 times the chances of using iodized salt as compared to youth household heads (AOR = 1.20, 95% CI = 1.12,1.28), 36–45 years (AOR = 1.16, 95% CI = 1.09,1.24), and more than 45 years (AOR = 1.18, 95% CI = 1.11,1.25). Households with lower and middle wealth indexes were 11% and 3% less likely, respectively, to not use iodized salt than richest households (AOR = 0.89, 95% CI = 0.76,0.89) and (AOR = 0.97, 95% CI = 0.81,0.93). Households with media exposure had 1.10 times the odds of using iodized salt compared to those with no media exposure (AOR = 1.10, 95% CI = 1.07–1.14). Households led by female household leaders had 1.08 times the odds of using iodized salt as those led by male household heads (AOR = 1.08, 95% CI = 1.04–1.12). Households with access to improved drinking water and better toilet facility types had 2.26, and 1.50-times higher odds of using iodized salt, respectively, than those with unimproved water and toilet (AOR = 2.26, 95% CI = 2.18–2.35) and (AOR = 1.50, 95% CI = 1.44,1.56). Households with more than five family members are less likely to use iodized household salt than households with fewer than five family members (AOR = 0.96, 95% CI = 0.93–0.99). Finally, regarding the community-level variables, households with high community wealth and high illiteracy had 1.54 and 0.60 times greater and lower probabilities of using iodized salt, respectively (AOR = 1.54, 95% CI = 1.27–1.87) and (AOR = 0.40, 95% CI = 0.33,0.49) (Table 3).

## Discussion

To achieve the long-term elimination of IDD, sufficient iodized salt must be made available and consumed. According to the WHO and International Council for Control of Iodine Deficiency Disorders (ICCIDD) standards, IDD can be eliminated if more than 90% of households consume adequately iodized salt. Despite the fact that salt iodization is the recommended low-cost technique for preventing iodine deficiency diseases [3], iodine deficiency disorders remain a severe public health problem in low-income countries [35, 36]. As a result, this study was carried out in order to identify the prevalence and associated factors of iodized salt use in East Africa, considering individual-, household-, and community-level factors. The final model revealed that household head’s age and gender, media exposure, wealth index, community education, education level, source of drinking water, toilet facility, number of family members, and community wealth were all substantially related to iodized salt use.

This study found that households with secondary/higher education had a 1.23 times chance of using iodized salt than those with no formal education. Furthermore, in the community-level variables, households with high illiteracy had 0.60 times lower probabilities of using iodized salt than literate communities. Education level is a key factor associated with the use of iodized salt. This study’s findings are congruent with those of other studies [7, 37, 38]. This increased likelihood of using iodized salt among educated participants could be attributed to better social interaction, knowledge, attitude, and awareness of the importance of utilizing iodized salt [39, 40].

Households with lower and middle wealth indexes were 11% and 3% less likely, respectively, to not use iodized salt than richest households. Similarly, regarding to community level variables, households with high community wealth index had shown more times greater probabilities of using iodized salt as compared to low wealthy communities. In this study, the wealth index is connected with the usage of iodized salt, which is consistent with previous studies conducted in low-income countries [7, 37, 38, 41]. When compared to households with the highest wealth position, those with lower and moderate wealth status had a lower chance of using iodized salt. Furthermore, compared to their peers, households from low neighborhood poverty levels were more likely to utilize iodized salt. This is due to the fact that household access to iodized salt is frequently based on awareness, availability, and price. In comparison to poor homes, high-income households can afford to buy iodine-containing salt from appropriate sources [22, 42]. Although, the author did not find other similar findings from the existing literatures, in this study, households with more than five members had a lower prevalence of iodization household salt usage. Similarly, this could be linked to less exposure to family planning, being less educated, coming from rural areas, and having a low household income. For instance, a study from Ethiopia showed that women with low education level, being rural residence, low economic status, and having no work have associated with large family size [43, 44].

Households with ages of 25–35 years, 36–45 years, and more than 45 years had 1.20, 1.16, and 1.18 times the chances of using iodized salt as compared to youth household heads, and more than 45 years. Older generations were up at an era when iodine deficiency was a major health problem. They may be more aware of the necessity of iodine in their diet and opt for iodized salt to achieve their iodine requirements [45]. Similarly, older persons may be more vulnerable to thyroid-related health problems, such as hypothyroidism or goiter, which can be caused by an iodine deficit [46, 47]. Iodized salt can help patients better prevent or manage certain illnesses. Older people may have had a lifelong habit of using iodized salt and continue to do so out of habit [27, 46]. Younger generations, on the other hand, may have embraced alternate salts or more natural alternatives as part of a health-conscious diet. Finally, iodized salt is regarded a staple ingredient in some cultures and has been passed down through centuries. Because of cultural customs, older people may have inherited this inclination and continue to use iodized salt.

Households with media exposure had 1.10 times the odds of using iodized salt compared to those with no media exposure. Similar to other studies [37, 40, 48], in this study, households with media exposure had a higher likelihood of using iodized salt than their counterparts. This is because media exposure is one of the most effective ways to raise people’s awareness and use of iodized salt [39, 40]. This study underlined the importance of using media in a consistent and effective manner to optimize the usage of iodized salt in the region.

Households led by female household leaders had 1.08 times the odds of using iodized salt as those led by male household heads. This finding might be related with female empowerment in the household responsibility. According to studies, women appear to bear more responsibility for household decisions more concerned about iodized salt than males), which is why microcredits are heavily concentrated on female borrowers in Bangladesh [22, 49, 50]. It could also be due to the fact that in Bangladesh, historically, females cook the food for the family and are more concerned about hygiene, and nutrition [51].

Households with access to improved drinking water and better toilet facility types had 2.26, and 1.50-times higher odds of using iodized salt, respectively, than those with unimproved water and toilet. Although, the author did not find earlier evidences regarding these factors, however some possible explanations can be justified. Safe and clean drinking water is crucial for general health and well-being, and improved water drinking can have an impact on it [52]. People are more likely to consume water from trustworthy and treated sources when they have access to improved water sources. Drinking iodized water can help boost iodine consumption, particularly in locations where iodine insufficiency is prevalent [53]. Promoting better water consumption increases the likelihood that people will consume iodized salt, which can eventually help resolve iodine deficits [52, 53]. Likewise, sanitation and basic hygiene habits are critical for sustaining health and reducing the spread of illness. Access to improved toilet facilities ensures proper waste disposal and reduces water contamination. Water contamination caused by poor sanitation can have a negative impact on iodine levels, and communities can protect the iodine content of salt by providing reliable and clean bathroom facilities.

### Strengths and limitations of the study

This study has several strengths. The first is the use of extremely recent, large, and representative datasets of East African countries obtained by well-trained data collectors using standard and validated questionnaires, which allow the study’s findings to be generalizable to the region. The second strength of this study is the use of multilevel modeling, a technique that accounts for the nested/hierarchical character of demographic and health surveys. Third, the study was trained to add new variables that may have an effect on the outcome variables, such as the source of drinking water, toilet facility, and women’s empowerment (as a household head), which were not included in prior studies. However, our study has several limitations. This study used quick test kits to determine whether households use iodine-containing salts. As a result, we were unable to quantify the amount of iodine in the salt and, as a result, we were unable to classify the use of iodized salt as appropriate or inadequate or to utilize any multiple classification methodologies. We were also unable to examine individual knowledge and food sources of iodine with the use of iodized salt due to the secondary nature of the study. Cultural diversity, availability, and country-level policy commitments may have impacted our results.

## Conclusions

According to the universal household iodization coverage plan, household iodized salt utilization in East Africa is moderately good. Both individual- and community-level characteristics were associated with the use of iodine-containing salt in East Africa. The final model revealed that household head age and sex of the household head, media exposure, wealth index, community education, individual education level, source of drinking water, toilet type, number of family members, and community wealth were all substantially related to iodized salt consumption. To boost the use of iodized salt in the region, there is a need to increase access to improved drinking water and toilet facilities, women’s empowerment, and participating male household leaders. Family planning, media sources, and supporting economically disadvantaged segments of the population will have a positive impact on the utilization of household iodized salts in Esat Africa.

## Data Availability

All data concerning this study are accommodated and presented in this document. The detailed data set can be freely accessible from the www.dhsprogram.comwebsite.
